# Supplementary material for: Perceived Occupational Noise Exposure and Depression in Young Finnish Adults
Source: Int J Environ Res Public Health. 2023 Mar 9;20(6):4850. doi: 10.3390/ijerph20064850 (PMC10049187; doi:10.3390/ijerph20064850)
Supplement: Supplementary file 1 [file ijerph-20-04850-s001.zip › FT12_17 year-old twin's questionnaire.pdf]

UNIVERSITY OF HELSINKI UNIVERSITY OF JYVÄSKYLÄ

LONGITUDINAL STUDY OF HEALTH AND BEHAVIOR IN TWIN CHILDREN

17 year-old twin's questionnaire

DEAR TWIN:

This questionnaire is part of medical research studying the influence of and relationship between genetic inheritance and environment on health and factors influencing health.

Now we are asking you to participate in our study; your role would be to answer the questions in this questionnaire. From a scientific perspective, it is important that as many of those who receive this questionnaire as possible answer it. The forms have been numbered for data processing purposes. Thus we will avoid sending a new form to those who have already answered. If your parents would like to see this form, we would hope they would do so before you answer the questions in it. For the reliability of the study it is very important that you answer the questions independently.

Be thorough in answering the questions. We hope you would find the time to complete the questionnaire within seven days.
Return the completed questionnaire in the enclosed return envelope. You can drop it at the post office without a stamp. We will pay for the postage.

NOTICE: YOUR IDENTITY AND THE INFORMATION YOU GIVE WILL REMAIN COMPLETELY CONFIDENTIAL. The results of the research will be studied in the form of tables compiled from all the answers, and won't reveal answers given by individual participants.

HOW TO ANSWER:

It is easy to fill out the questionnaire. You will always answer each question or sub-question by circling one of the alternatives. The alternatives have been numbered.

Some of the questions have, after the answer alternatives, a supplementary question or an indication to move on to a certain other question. In some of the questions you are asked to write or number something.

If you have to correct an answer, cross out the faulty answer.

Background

1. What is your first name?________________________________

2. In addition to you and your siblings, does your family consist of

   1 mother and father
   2 mother and stepfather
   3 father and stepmother
   4 only mother
   5 only father
   6 other, who? ___________________________

Education and work

3. Are you presently attending school or studying?

   1 I don't go to school nor do I study
   2 I go to school or study, I don't work
   3 I go to school or study, but also work for _______ hours / week

4. What is your level of education so far? (Circle 1 = 'yes ' or 2 = 'no' for every alternative)

	kyllä	ei
Comprehensive school, certificate	1	2
Comprehensive school, uncompleted	1	2
Comprehensive school, 10th grade	1	2
[ylioppilastutkinto, roughly equivalent to high school plus first 2 years of college, and a national exam, at age 17 - 19]	1	2
I have completed an apprenticeship contract,	1	2
what line : ____		
I have completed a vocational training course or a job training,	1	2
what line: ____		

5. What kind of education are you attending at present? (If you do not study, answer the next question).

1 Comprehensive school, 10th grade

2 High school

3 I am in a vocational school, what line?

____

4 I have an apprenticeship contract, what line? ____________________

____

5 I am attending a vocational training course or job training, what line _______

____

6 I am in a polytechnic school or college; what line? ________________

____

7 Something else, what is it, and what line? ______________________

____

6. If you don't presently study or attend school, what do you do?
I am:

1 in temporary training in order to continue my studies

2 working _______ hours / week

3 unemployed or on forced leave

4 at home

5 other, what? ___________________________________

Home and family

7. How many times have you moved to another location since 6th grade? _____ times.

1 I have not moved since 6th grade → go to question 9.

8. Did you move

1 from a place of larger population to a smaller place.

2 from a place of smaller population to a larger place.

3 to a place of similar size.

9. How many different schools have you attended since 6. grade? ________ schools.

10. How do the following features describe the atmosphere at your home? Evaluate how well each statement describes your home.

	not at all	only little	somewhat	quite well	completely
Warm, caring	1	2	3	4	5
Encouraging, supporting	1	2	3	4	5
Trusting, understanding	1	2	3	4	5
Open	1	2	3	4	5
Hard, punishing	1	2	3	4	5
Unfair	1	2	3	4	5
Quarrelsome	1	2	3	4	5
Indifferent	1	2	3	4	5

11. I have weekly __________ mk money for myself.

12. My parents know how I use my money

1 very well

2 quite well

3 quite poorly

4 very poorly

Leasure time

13. How much time do you use/how often you do the following things? Please give an answer for each activity.

	I don't	Once in six months	Once in a month	Once per week	A few times per week	Daily
watching TV	0	1	2	3	4	5
watching videos	0	1	2	3	4	5
playing video/computer games	0	1	2	3	4	5

	I don't	Once in six months	Once in a month	Once per week	A few times per week	Daily
playing some musical instrument	0	1	2	3	4	5
reading (other than studying)	0	1	2	3	4	5
drawing or painting pictures	0	1	2	3	4	5
doing handicrafts or building something (e.g. scale models)	0	1	2	3	4	5
spending time with my friends in town/at the village/ places where adolescents meet	0	1	2	3	4	5
going to the movies	0	1	2	3	4	5
going to theatre/concerts (rock/classical music)	0	1	2	3	4	5
dancing/discotheques	0	1	2	3	4	5
going to some club, or to some other organized activity	0	1	2	3	4	5
going to the bar	0	1	2	3	4	5
participation in church or other religious activities	0	1	2	3	4	5

14. How often do you exercise or do sports during your free time? (school physical activities don't count here).

1 not at all → pass on to question 16.

2 less than once a month

3 1 – 2 times a month

4 about once a week

5 2 – 3 times a week

6 4 – 5 times a week

7 just about every day

15. Which of the following alternaties best describes your present sports/fitness activities? I usually do sports or exercise so that:

1 I breathe hard and sweat profusely

2 I breathe rather hard and sweat somewhat

3 I don't breathe very hard and sweat but little

4 I don't sweat or breathe hard

16. How do you perceive your present physical condition? Is it

1 very good

2 rather good

3 satisfactory

4 rather poor

5 very poor

17. Have you got a computer available at home

1 no, I have not

2 yes, a computer without Internet connection

3 yes, a computer with Internet connection

18. How much time do you spend with the computer (0 = not at all or seldom)

leasure time: ________ hours a week (on the average)

work or studying: _______ hours a week (on the average)

19. How much is your monthly mobile phone bill?

1 I haven't got a mobile phone

2 less than 50 mk/month

3 50 - 99 mk/month

4 100 - 199 mk/month

5 200 - 499 mk/month

6 500 mk/month or more

20. How many e-mail messages or mobile phone text messages do you send on the average?

1 not any e-mail or mobile phone messages

2 a message once a week or less

3 a few messages a week, but not every day

4 1 – 4 messages a day

5 5 – 9 messages a day

6 10 or more messages a day, how many? _________

21. Do you send e-mail or mobile phone text messages to your twin? (please answer both the amount of e-mails and text messages, if you do)

1 I do not send e-mails or text messages at all to my twin

2 I send text messages, how many per week? _________

3 I send e-mails, how many per week? ________

Growth and Development

22. How tall are you? ____________ cm.

23. How much do you weigh? _________ kg.

24. The next question about pubertal development is different for boys and girls:

Girls answer: How old were you when you first menstruated?

___________ years old

1 I have not begun to menstruate yet

Boys answer: How old were you when your voice changed?

___________ years old

1 my voice has not changed yet

25. Comparing yourself with other youths of your age and sex, would you say that your growth in heigh and puberty has been

1 clearly more advanced than theirs

2 somewhat more advanced than theirs

3 average development

4 somewhat less advanced than theirs

5 clearly less advanced than theirs

26. Comparing yourself with your co-twin, would you say that your growth in heigh and puberty has been

1 clearly more advanced than his/her

2 somewhat more advanced than his/her

3 the same

4 somewhat less advanced than his/her

5 clearly less advanced than his/her

Health

27. How do you view your health? Is it presently

1 very good

2 rather good

3 mediocre

4 rather poor

5 very poor

28. How often have you had the following symptoms during the past month?

	seldom or never	about once a mounth	about once a week	more than once a week	almost daily
headache	1	2	3	4	5
stomach ache	1	2	3	4	5
depression	1	2	3	4	5
difficulties sleeping	1	2	3	4	5
tiredness during daytime	1	2	3	4	5
waking up during nights	1	2	3	4	5
tension or nervousness	1	2	3	4	5
irritability or fits of anger	1	2	3	4	5
back or neck pains	1	2	3	4	5

29. In the last three years, has there been any substantial changes in your health (for example, a serious illness or an accident)? If yes, explain in more detail

____

____

____

30. Please tell us what you think about each of these statements. Check the alternative that best describes what you think.

	strongly disagree	partly disagree	difficult to say	partly agree	strongly agree
A moderate use of alcohol is part of normal life.	1	2	3	4	5
Trying marijuana or hashish is no more dangerous than drinking a few bottles of beer.	1	2	3	4	5
It's fair that the law prohibits the sale of beer to persons under 18 years of age.	1	2	3	4	5
A drink every now and then merely acts as a pick-me-up and cannot be considered out of place.	1	2	3	4	5
Kids that use alcohol are more popular among peers.	1	2	3	4	5
When you've had a drink, it's easier to get acquainted with the opposite sex.	1	2	3	4	5
There is too much fuss about the dangers of smoking nowadays.	1	2	3	4	5
It is sometimes the case that a genuine, spontaneous relaxed atmosphere is only born when alcohol is used.	1	2	3	4	5
It is fair that the law prohibits the sale of tobacco to persons under 18 years of age.	1	2	3	4	5
It is important that one should be able to drink oneself into a state of intoxication in order to relax and say what one has to say.	1	2	3	4	5

Smoking

31. Have you ever smoked (or tried smoking, at least one cigarette)?

1 no → go to question 36

2 yes

32. How old were you when you first tried smoking?

__________ years old

33. How many cigarettes have you smoked altogether up to now?

1 only one

2 about 2 - 10

3 about 11 - 50

4 about 51 – 100

5 over 100

34. Which of the following best describes your present smoking habits?

1 I smoke 20 cigarettes or more each day

2 I smoke 10 – 19 cigarettes each day

3 I smoke 1 – 9 cigarettes each day

4 I smoke once or more a week, but not every day

5 I smoke less often than once a week

6 I am trying to or have quit smoking

7 I have tried smoking but I don't smoke

35. How many times have you tried to quit smoking?

1 I have not tried to quit

2 once

3 2 – 5 times

4 over 5 times

36. Do any of your friends smoke?

1 no one smokes

2 one of them smokes

3 2-5 of them smoke

4 more than 5 of them smoke

37. Have you ever tried snuff? How many times altogether up to now?

1 no
2 I have tried once
3 I have taken snuff 2 - 50 times
4 I have taken snuff over 50 times
5 I take snuff regularly

Alcohol and drug use

38. Do any of your friends drink alcohol regularly?

1 no one drinks
2 one of them drinks
3 2-5 of them drink
4 more than 5 of them drink

39. How often do you drink alcohol at all? Try to include the times also when you drink very small quantities, like just half a bottle of light beer or a sip of wine.

1 daily
2 couple of times a week
3 once a week
4 a couple of times a month
5 about once a month
6 about once every two months
7 2 – 4 times a year
8 once a year or less
9 I don't drink any alcohol

40. How often do you drink so that you get at least slightly intoxicated?

1 daily
2 couple of times a week
3 once a week

4 a couple of times a month

5 about once a month

6 about once every two months

7 2 – 4 times a year

8 once a year or less

9 I don't drink any alcohol

41. On how many days total have you had beer, wine, or liquor during the past four weeks?

on _______ days

0 I didn't drink at all

42. If I would drink so much alcohol that I would feel its effect, I would become: (answer to each question how alcohol would affect you)

	never	sometimes	often	don't know
sleepy	1	2	3	4
talkative	1	2	3	4
sad	1	2	3	4
angry	1	2	3	4
ill feeling	1	2	3	4
friendly	1	2	3	4
confused	1	2	3	4
mean	1	2	3	4
content	1	2	3	4
fun	1	2	3	4
depressed	1	2	3	4

43. Do you know anyone among your acquaintances who would have tried drugs (hashish, paint thinner or something else to sniff, prescription drugs, or other substances like that)?

1 I don't know any adolescents who have done that

2 I know one

3 I know 2 – 5

4 I know more than 5 adolescents who have done that

44. And you? Have you tried drugs (hashish, paint thinner or something else to sniff, prescription drugs, or other substances like that)?

1 I have not

2 1 - 3 times

3 4 - 9 times

4 10 - 19 times

5 > 20 times

Opinions and feelings (?)

45. The next questions deal with your opinions, attitudes and feelings.Your task is to choose the alternative that best describes your **usual way** to behave or feel – not how you feel at the moment, but what kind of person you usually are. Do it quickly – do not consider it too long.

	strongly disagree	disagree	agree	strongly agree
I have had more than my share of things to worry about.	1	2	3	4
I have a tendency to act on the spur of the moment without really thinking ahead	1	2	3	4
I often find myself gnashing my jaws together, for no real reason.	1	2	3	4
My parents have often disapproved of my friends.	1	2	3	4
When I have to make a decision, I "sleep on it" before I decide.	1	2	3	4
Life usually hands me a pretty raw material.	1	2	3	4
I usually get so excited over new ideas and suggestions that I forget to check if there are any disadvantages.	1	2	3	4
My home life was always very pleasant.	1	2	3	4
My body often feels stiff and tense.	1	2	3	4
As a child I have often gone against my parents' wishes.	1	2	3	4
When trying to fall asleep I often notice that my muscles are really tense.	1	2	3	4
I often throw myself too hastily into things.	1	2	3	4
An unexpected noise makes me jump and startle.	1	2	3	4
The members of my family were always very close to each other.	1	2	3	4

	strongly disagree	disagree	agree	strongly agree
Often I find myself holding the newspaper tightly, when I am reading it.	1	2	3	4
In school I was sometimes sent up to the principal for cutting up.	1	2	3	4
I sometimes wanted to run away from home.	1	2	3	4
I usually "talk before I think".	1	2	3	4
I have difficulty sitting in a relaxed position even in a comfortable chair.	1	2	3	4
As a youngster in school I used to give the teacher lots of trouble.	1	2	3	4
My hands usually tremble.	1	2	3	4
When I am about to make a decision I usually make it quickly.	1	2	3	4
My parents never really understood me.	1	2	3	4.
My muscles are so tense that I get tired.	1	2	3	4
When I was going to school I played hooky quite often.	1	2	3	4
I consider myself an impulsive person.	1	2	3	4
In the late afternoon I often get a headache which feels as if there were and iron-band across my forehead.	1	2	3	4

46. Choose the best fitting alternative

	strongly disagree	disagree	agree	strongly agree
On the whole, I am satisfied with myself.	1	2	3	4
At times I think I am no good at all.	1	2	3	4
I feel that I have a number of good qualities.	1	2	3	4
I am able to do things as well as most other people.	1	2	3	4
I feel I do not have much to be proud of.	1	2	3	4
I certainly feel useless at times.	1	2	3	4
I feel that I'm a person of worth, at least on an equal basis with others.	1	2	3	4
I wish I would have more respect for myself.	1	2	3	4
All in all, I am inclined to feel that I am a failure.	1	2	3	4
I take a positive attitude toward myself.	1	2	3	4

I do as I see fit, nevermind what my friends try to lure me into	1	2	3	4

47. On school/workday mornings I tend to be

    1 quite awake

    2 somewhat awake and rested

    3 fairly tired

    4 extremely tired

48. How long time does it take for you to get started in the morning after a night's sleeping?

    1 about 10 minutes or less

    2 more than 10 minutes, but less than 20

    3 more than 20 minutes but less than 40

    4 more than 40 minutes

49. Try to estimate your "morningness" or "eveningness"

    1 I'm obviously a morning person (early riser and get sleepy towards evening)

    2 I'm somewhat a morning person

    3 I'm somewhat an evening person (sleepy in the morning and fresh in the evening)

    4 I'm obviously an evening person

Sensations

50. Answer the following questions. Choose the best fitting alternative.

	never	sometimes	often	very often
Have you become sad, depressed, or irritable for several days or more without really understanding why?	1	2	3	4
Have there been periods of time when you lost almost all interest in the things that you usually like to do (such as hobbies, school work, entertainment)?	1	2	3	4
Have there been periods lasting several days or more when you spent much of your time brooding about upleasant things that have happened?	1	2	3	4
Have you had periods when you were so down that you found it hard to start talking or that talking	1	2	3	4

took too much energy?

	never	sometimes	often	very often
Have you experienced several days or more when you were feeling down and depressed, and you also were physically restless, unable to sit still, and had to keep moving or jumping from one activity to another?	1	2	3	4
Have you had periods of sadness and depression when, for several days or more, it took you over an hour to get sleep at night, even though you were very tired?	1	2	3	4
Have there been times of several days or more when you really got down on yourself and felt worthless?	1	2	3	4
Have there been periods of several days or more when you were slowed down and couldn't move as quickly as usual?	1	2	3	4
Have you had long periods when you were down and depressed, interrupted by brief periods when your mood was normal or slightly happy?	1	2	3	4
Have there been periods of time when you felt a persistent sense of gloom?	1	2	3	4

Friends and leisure time

51. Are you dating regularly at present?

    1 no, I'm not

    2 yes, I am

52. With whom do you usually spend your free time?

    1 alone

    2 with my family

    3 with my boyfriend/girlfriend

    4 with one friend

    5 with two of my friends

    6 with a larger group

53. How much of your free time do you usually spend with your twin?

    1 I spend almost all of my free time with my twin

2 I spend a little bit more of my free time with my twin than with others

3 I spend a little bit more of my free time with others than with my twin

4 I spend most of my free time with others than my twin

5 We live apart, we cannot spend time together

54. Estimate how well the following descriptions fit you:

	not at all	sometimes	rather well	well
I am a good leader.	1	2	3	4
I am friendly to others.	1	2	3	4
I am restless and can't sit still.	1	2	3	4
I am calm and patient.	1	2	3	4
I act before thinking.	1	2	3	4
I try to solve difficult problems reasonably, and consider other people.	1	2	3	4
I am unable to concentrate on anything.	1	2	3	4
When I am mad at someone I sometimes decide to exclude him/her.	1	2	3	4
I usually do not feel at ease when I meet people I do not know too well.	1	2	3	4
I sort out things through discussion.	1	2	3	4
I sometimes feel the desire to tease, to annoy, or to attack another person without reason.	1	2	3	4
I talk all the time.	1	2	3	4
I defend those who are weaker.	1	2	3	4
I do not listen to directions.	1	2	3	4
I spread rumors about other people's personal matters when I'm mad at them.	1	2	3	4
I'm scared by or nervous about new things and situations	1	2	3	4
I'm a person everyone can trust.	1	2	3	4
Given enough provocation, I may hit another person.	1	2	3	4
I'm very energetic, always on the go and often have contact with other people.	1	2	3	4
I avoid difficult situations by doing something else.	1	2	3	4
My moods change often, and I lose my temper easily.	1	2	3	4

I help others when they need it.	1	2	3	4

	not at all	sometimes	rather well	well
If someone annoys me, I am apt to tell him what I think of him.	1	2	3	4
I'm too impatient to wait for my turn.	1	2	3	4
I give up easily and behave according to expectations.	1	2	3	4
I'm popular among other youths.	1	2	3	4
I am the kind of person who is excessively sensitive and easily hurt.	1	2	3	4
I always do my tasks.	1	2	3	4
I'm reliable and stable. I keep my composure in all situations.	1	2	3	4
I am often teased.	1	2	3	4
It takes me an unusually long time to get over unpleasent events.	1	2	3	4
I forget things.	1	2	3	4
I often become angry, and I easily get involved in quarrels or fights.	1	2	3	4
I am quiet, withdrawn, and often alone.	1	2	3	4
Even though I know I am right, I often have great difficulty getting my points across.	1	2	3	4
When people yell at me, I yell back.	1	2	3	4
I am hyperactive.	1	2	3	4

55. Next, estimate how well the following descriptions fit to your **twin**:

	not at all	sometimes	rather well	well
My twin is a good leader.	1	2	3	4
My twin is always friendly to others.	1	2	3	4
My twin is restless and can't sit still.	1	2	3	4
My twin is calm and patient.	1	2	3	4
My twin acts before thinking.	1	2	3	4
My twin tries to solve difficult problems reasonably, and consider other people.	1	2	3	4
My twin is unable to concentrate on anything.	1	2	3	4

	not at all	sometimes	rather well	well
When my twin is mad at someone he/she sometimes decides to exclude this person.	1	2	3	4
My twin usually does not feel at ease when he/she meets people he/she does not know too well.	1	2	3	4
My twin sorts out things through discussion.	1	2	3	4
My twin sometimes feels the desire to tease, to annoy, or to attack another person without reason.	1	2	3	4
My twin talks all the time.	1	2	3	4
My twin defends those who are weaker.	1	2	3	4
My twin does not listen to directions.	1	2	3	4
My twin spreads rumors about other people's personal matters when he/she is mad at them.	1	2	3	4
My twin is scared by or nervous about new things and situations.	1	2	3	4
My twin is a person everyone can trust.	1	2	3	4
Given enough provocation, my twin may hit another person.	1	2	3	4
My twin is very energetic, always on the go and often has contact with other people.	1	2	3	4
My twin avoids difficult situations by doing something else.	1	2	3	4
My twin's moods change often, and he/she loses his/her temper easily.	1	2	3	4
My twin helps others when they need it.	1	2	3	4
If someone annoys my twin, my twin is apt to tell this person what he/she thinks of him.	1	2	3	4
My twin is too impatient to wait for his/her turn.	1	2	3	4
My twin gives up easily and behaves according to expectations.	1	2	3	4
My twin is popular among other youths.	1	2	3	4
My twin is the kind of person who is excessively sensitive and easily hurt.	1	2	3	4
My twin always does his/her tasks.	1	2	3	4
My twin is reliable and stable. My twin keeps his/her composure in all situations.	1	2	3	4

My twin is often teased.	1	2	3	4

	not at all	sometimes	rather well	well
It takes him/her an unusually long time to get over unpleasent events.	1	2	3	4
My twin forgets things.	1	2	3	4
My twin often becomes angry, and he/she easily gets involved in quarrels or fights.	1	2	3	4
My twin is quiet, withdrawn, and often alone.	1	2	3	4
Even though my twin knows he/she is right, he/she often has great difficulty getting his/her points across.	1	2	3	4
When people yell at my twin, he/she yells back.	1	2	3	4
My twin is hyperactive.	1	2	3	4

56. Are you satisfied with your

	not at all	mainly no	somewhat	mainly yes	completely
spending leisure time at home	1	2	3	4	5
spending leisure time outside the home	1	2	3	4	5
success at work/studies	1	2	3	4	5
relationship with your mother	1	2	3	4	5
relationship with your father	1	2	3	4	5
relationship with your twin	1	2	3	4	5

In the following space you can provide additional information if our questions have not addressed some aspect of your health.

____

____

____

It may become necessary to ask further questions later. We hope that if the need arises, we could contact you by telephone.

My telephone number is (_____) _______________________

As we told you in the letter inclosed, we will give your personality characterization on the ground of this questionnaire. If you **do not wish to have** this kind of feedback, please mark it here for our information.

☐ no thanks, I do not want any feedback.

! **We would kindly request you to make sure that you have answered all the questions.**

We thank you for your assistance in this medical study.
