# Supplementary material for: Perceived Occupational Noise Exposure and Depression in Young Finnish Adults
Source: Int J Environ Res Public Health. 2023 Mar 9;20(6):4850. doi: 10.3390/ijerph20064850 (PMC10049187; doi:10.3390/ijerph20064850)
Supplement: Supplementary file 1 [file ijerph-20-04850-s001.zip › FT12_21-24y_Homequestionnaire Intensive sample.pdf]

UNIVERSITY OF HELSINKI
DEPARTMENT OF PUBLIC HEALTH

FT12 STUDY / THE FOURTH WAVE OF DATA COLLECTION

QUESTIONNAIRE FOR TWINS AT THE AGE OF 20-24

DEAR RECIPIENT

This questionnaire is a part of a medical research project to study the environmental and genetic effects on young adults' health and factors affecting them. You have participated in it earlier at the ages of 11, 12, 14 and 17.

We now kindly ask You to reply to this questionnaire. Questionnaires are numbered for computer processes. For the sake of the study's reliability it is important that You answer to the questions independently.

Please answer carefully. A **Please check finally that there are no questions left unanswered by mistake.**

NOTE: YOUR ID AND THE INFORMATION YOU HAVE GIVEN US IS KEPT IN COMPLET SECRECY. All the given answers are analyzed in table formats and no individual responses can be identified by them.

ANSWER LIKE THIS:

Filling the questionnaire is easy. Answer to each question or sub-question by drawing a circle around one alternative. The alternatives are numbered. If none of the alternatives suits You, choose the one that suits You the best.

After some questions there is a sub-question or a request to skip to some other question. In some questions You are asked something You have to write down or where you have to circle a number.

In case you have to correct your answer, just draw a cross over the incorrect answer.

Before the actual questionnaire, please write down the date of filling this questionnaire:

____ / _____ 2009.

1. What is your first name? ___________________________________

Study and work

2. Do you go to school or are you studying at the moment?

1 no, I am not studying anywhere
2 I'm studying, not working at the same time
3 I'm studying and also working ______ hours per week

3. What schools/degrees have you completed?
(Circle 1="yes" or 2= "no" to each alternative)

	yes	no
A) Senior grades of the basic school	1	2
B) I have a degree from the 10th class of the basic school	1	2
C) I have passed my matriculation examination	1	2
D) I have a vocational degree	1	2
E) I have a polytechnic degree	1	2
F) I have an academic degree	1	2
G) I have passed an apprenticeship training	1	2
H) I have passed a vocational training or vocational course	1	2

Use of (leisure) time

4. How much time do you use or how often do you do the following things? Estimate each item separately. Circle the best alternative.

		**less than once a month**	**about once a month**	**about once a week**	**twice a week**	**daily less than 2 hours per day**	**daily over 2 hours**
1	watching TV	1	2	3	4	5	6
2	watching videos	1	2	3	4	5	6
3	PC- and TV- games	1	2	3	4	5	6
4	playing any instrument	1	2	3	4	5	6
5	reading (other than studying)	1	2	3	4	5	6
6	drawing, painting	1	2	3	4	5	6

7	handicraft, doing joinery, constructing scale models	1	2	3	4	5	6
8	informal get-togethers at the city/village/or other places where young people tend to gather	1	2	3	4	5	6
9	going to movies	1	2	3	4	5	6
10	going to dances or disco	1	2	3	4	5	6
11	taking part in clubs or other organizational activities	1	2	3	4	5	6
12	going to fast-food restaurants	1	2	3	4	5	6
13	going to bars	1	2	3	4	5	6
14	taking part in activities arranged by church or other religious	1	2	3	4	5	6

5. With whom would you rather spend your leisure time?

1 by myself
2 with family
3 with the person I go steady
4 with one friend
5 with two friends
6 with a bigger group of people

Physical activity

6. What do you think about your current physical condition? Is it

1 very good
2 quite good
3 satisfactory
4 quite poor
5 very poor

7. How often do you use any vehicle (car, bus, train e.g.)?

1 monthly
2 weekly
3 daily less than 15 minutes
4 daily 15 to 29 minutes
5 daily 30 to 59 minutes
6 daily 1 to 2 hours
7 daily over 2 hours

8. Is there a car easily available for you?

1 yes
2 no

9. How much time altogether of your daily way to work or student place is spent by walking, cycling and/or cross-country skiing?

1 less than 15 minutes
2 15 to 29 minutes
3 30 to 59 minutes
4 an hour or more
5 I'm not working or studying

10. How often do you go in for sports or take physical exercise during your leisure time?

1 not at all
2 less than once a month
3 1 to 2 times a month
4 about once a week
5 2 to 3 times a week
6 4 to 5 times a week
7 nearly every day
8 many times a day

11. Is your leisure time exercise much the same strength demanding as:

1 walking
2 alternatively walking and light run
3 light run (jogging)
4 running

12. On an average how long does one set of leisure time physical exercise last?

1 less than half an hour
2 half an hour – less than an hour
3 an hour - less than 2 hours
4 2 hours or longer

13. On an average how much time per day do you spend on physically demanding leisure time activities? (e.g. gardening, repair work, cleaning) Do not count work, way to work or leisure time exercise.

1 less than half an hour
2 half an hour – less than an hour
3 an hour – less than 2 hours
4 2 hours or more

14. On an average how much time per day do you spend on leisure time activities which are not physically demanding? (e.g. craft, reading, watching TV, playing computer games, surfing at internet, sitting in car)

1 less than half an hour
2 half an hour – less than an hour
3 an hour – less 2 hours
4 2 hours – 4 hours
5 over 4 hours

15. Are you content with the size of your muscles?

1 always
2 usually
3 often
4 sometimes
5 seldom
6 never

16. Have you ever used any hormones to increase your muscle mass or to make your workout (at the gym) more effective?

1 yes, repeatedly within the past 3 months
2 yes, in the past repeatedly within 3 or more months
3 I have tried sometimes
4 never

17. Have you ever used any supplements or other special products to increase your muscle mass or to make your workout (at the gym) more effective?

1 yes, repeatedly within the past 3 months
2 yes, in the past repeatedly within 3 or more months
3 I have tried sometimes
4 never

Growth and development processes

18. How tall are you? __________cm

19. What is your current weight? _________kg (Women note: if you are pregnant at the moment code your weight before pregnancy)

20. For how long have you weighted your current weight? ____year ____mo (E.g. your cloth size has remained the same. Women: don’t count the time of pregnancy or breast feeding)

21. What do you regard as your ideal weight? __________kg

22. When thinking back the past 12 months which of the following alternatives describes you the best?

1 I did not try to influence my weight at all

2 I tried to maintain my current weight

3 I tried to gain my weight

4 I tried to lose my weight

23. In your lifetime how many times have reduced your weight more than 5 kg?
(Women: do not count changes in weight due to pregnancy or breast feeding)

1 never

2 once

3 2 to 4 times

4 5 or more times

24. How old were you when you tried to reduce your weight for the first time?

1 ___________ years

2 I have never tried to reduce my weight

General health

25. During the past months how often have you had the following symptoms?

		Nearly every day	More often than once a week	About once a week	About once a month	More seldom or never
1	headache	1	2	3	4	5
2	stomachache	1	2	3	4	5
3	depression	1	2	3	4	5
4	difficulties in falling asleep	1	2	3	4	5
5	feeling fatigue at daytime	1	2	3	4	5
6	waking up at night	1	2	3	4	5
7	tension and nervousness	1	2	3	4	5
8	please circle nb. 1, this is a checkpoint	1	2	3	4	5
9	irritability and bursts of anger	1	2	3	4	5

26. Think about the time from past 3 months till today. How often have you had pain or ache in the following part of your body (parts of the body are marked at the pictures above)? Circle the appropriate alternative at the table below.

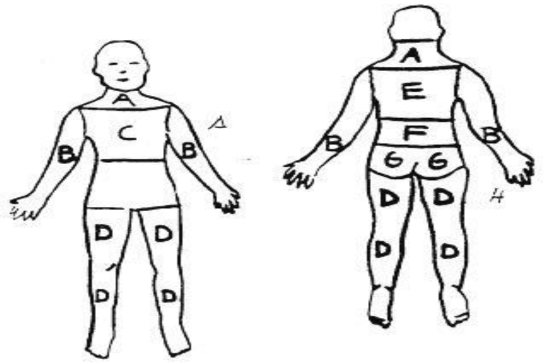

		Nearly every day	More often than once a week	About once a week	About once a month	More seldom or never
1	Neck or shoulders (A)	1	2	3	4	5
2	Upper limbs (hands) (B)	1	2	3	4	5
3	Chest (C)	1	2	3	4	5
4	Lower limbs (feet) (D)	1	2	3	4	5
5	Upper back (E)	1	2	3	4	5
6	Lower back (F)	1	2	3	4	5
7	Buttocks (G)	1	2	3	4	5

Smoking and use of alcohol

27. Which of the following alternatives describes your current smoking behavior the best?

1 I smoke daily 20 cigarettes or more
2 I smoke daily 10 to 19 cigarettes
3 I smoke daily 1 to 9 cigarettes
4 I smoke once a week or more often, not daily however
5 I smoke less frequently than once a week
6 I have stopped or quit smoking
7 I have tried smoking but I don’t smoke
8 I have never even tried

28. How often do you drink alcohol? Try to take into account also the times, when you drink very small amounts of alcohol, e.g. a half bottle of middle strength beer or a drop of wine.

1 daily
2 a couple of times a week

3 once a week
4 a couple of times a month
5 about once a month
6 about once in a couple of month
7 2 to 4 times a year
8 once a year or less often
9 I don’t drink alcohol

29. How often do you drink alcohol to the extent that you are really drunk?

1 daily
2 a couple of times a week
3 once a week
4 a couple of times a month
5 about once a month
6 about once in a couple of month
7 2 to 4 times a year
8 once a year or less often
9 I don’t drink alcohol

About religion

30. Do you regard that religion as a part of your life is

1 very important
2 important
3 not so important
4 not at all important
5 I don’t know

31. Do you go to church or other religious occasions (do not count weddings, funerals, confirmation or christening ceremonies)

1 once a week
2 about once a month
3 about once a year
4 less often
5 I don’t go to church

32. How important to you is your spouse’s or close friends’ religious conviction?

1 very important
2 important
3 not so important
4 not at all important
5 I have not thought about it

33. Please read the following arguments and decide **whether the argument suits you or not.** If regarding to you the argument is true or quite true circle the word **True** in front of the argument. If regarding to you the argument is **untrue** or is **not usually true,** circle the word **False** in front of the argument. If the argument does not suit you at all or you don't understand its meaning, don't circle either of the alternatives.

True False 1. Everything happens just like the prophets in the Bible have predicted
True False 2. I go to church nearly every week
True False 3. I believe in Christ's Second Coming
True False 4. I believe in life after death
True False 5. I'm very religious (more religious than most people)
True False 6. I believe that devil and hell exist in the life after death
True False 7. I believe that God exists
True False 8. I'm sure there is only one right religion
True False 9. Christ performed miracles e.g. changed water to wine
True False 10. I pray many times in a week
True False 11. I read Bible many times in a week
True False 12. I cannot stand people who think that there is only one right religion

Courtship

34. For how long has your relationship with your current partner lasted?

1 I am no going steady with somebody
2 I am going steady or I am married or living together with someone as though we were married. Our relationship began about _____years ago

35. How many sexual partners have you had?

1 none
2 one
3 two
4 three or four
5 five or more

36. At what age did you have sex for the first time?

age___________

Economic situation

37. Are you worried about yours or your family's:

	not at all	a bit	somewhat	quite much	very much
A) economic survival?	1	2	3	4	5
B) capability of getting food, clothes e.g.?	1	2	3	4	5

Goals

38. People have different kinds of goals, projects or plans. These personal goals can be related to different kinds of fields of life such as studying, friends, family, work, dating, health, own parents, purchases or spending money, travelling, oneself, or hobbies. **We ask you to write down on the lines below the 4 goals which are important for you at present.**

1. Goal 1:____

2. Goal 2:____

3. Goal 3:____

4. Goal 4: ____

Now write down on the line below one personal goal of yours related to STUDIES or CAREER (it may be the same you have already mentioned above).

5. ____

Now evaluate this goal of yours related to studies or career. Answer to each question or statement by drawing a circle around the number which corresponds your opinion the best. (The alternatives are from 1 to 7)

1= Very little…………7=Very much

6.	How **important** is that goal of yours?	1	2	3	4	5	6	7
7.	How **committed** are you to that goal?	1	2	3	4	5	6	7
8.	To what extent have you **proceeded** in achieving the goal?	1	2	3	4	5	6	7
9.	How much **time and effort have you spent** to achieve that goal?	1	2	3	4	5	6	7
10.	How **strength demanding and burdening** is your goal?	1	2	3	4	5	6	7
11.	What are the odds that your goal is to **come true**?	1	2	3	4	5	6	7
12.	To what extent are you aware of **what to do** to achieve your goal?	1	2	3	4	5	6	7

13.	How **capable** are you to achieve your goal?	1	2	3	4	5	6	7
14.	To what extent do you have **means** to achieve your goal?	1	2	3	4	5	6	7
15.	How much **work have you done** to achieve your goal?	1	2	3	4	5	6	7
16.	How **stressful** is your goal?	1	2	3	4	5	6	7

Working life

39. During the past 12 months have you been at work?

1 daily
2 weekly
3 monthly
4 temporary work
5 I have not worked at all or it has been very occasional (skip to item 52, page 17)

40. Altogether how many months have you been at work during the past 12 months?

____________ months (change the days/weeks to months)

41. What have your duties been like?

____

____

42. How do you consider your working capacity if you compare it to the physical demands of your current /the latest work? Is it:

1 very good
2 quite good
3 moderate
4 quite poor
5 very poor

43. How do you consider your working capacity if you compare it to the mental demands of your current /the latest work? Is it:

1 very good
2 quite good
3 moderate
4 quite poor
5 very poor

44. Here are some questions about work or working environment. Answer them regarding to the work which has been your main work during the past 12 months.

		**Fully agree**	**Somewhat agree**	**Not agree nor disagree**	**Somewhat disagree**	**Fully disagree**
1	I have to be quick at my work	1	2	3	4	5
2	My work demands very hard work	1	2	3	4	5
3	I am required an excessive amount of workload	1	2	3	4	5
4	I have enough time to finish my work	1	2	3	4	5
5	My work is very quick-paced	1	2	3	4	5
6	I can make many independent decisions at my work	1	2	3	4	5
7	My work presumes me creativeness	1	2	3	4	5
8	My work demands me to learn new things	1	2	3	4	5
9	My work consists of many repetitious things	1	2	3	4	5
10	I have much voice in my work	1	2	3	4	5
11	My work demands many skills at an advanced state	1	2	3	4	5
12	I am able to perform many various things at my work	1	2	3	4	5
13	I am able to develop the special talents characteristics to me	1	2	3	4	5
14	I have very few degrees of freedom to decide how I perform my work	1	2	3	4	5
15	I can get support from my closest superior when needed	1	2	3	4	5
16	I can get support from my co-workers when needed	1	2	3	4	5
17	We discuss together about duties, goals and how to reach them at my work unit	1	2	3	4	5
18	I’m content with the way my superior expresses the value of my work	1	2	3	4	5
19	I am able to punctuate my work with adequate amount of pauses	1	2	3	4	5
20	I am able to punctuate enough the length of my workday	1	2	3	4	5

45. The following statements deal with leadership at your work. Circle the alternative which describes your opinion at your work the best.

		**Fully agree**	**Quite agree**	**Not agree nor disagree**	**Quite disagree**	**Fully disagree**
1	At my work decisions are made based on adequate knowledge	1	2	3	4	5
2	Decisions made have been logical (the rules are the same for everyone)	1	2	3	4	5
3	Everyone has the right to express one's own opinion concerning things about his/herself	1	2	3	4	5
4	At my work unsuccessful decisions can be cancelled or they may be changed	1	2	3	4	5
5	In important matters our superior listens to the opinions of his/her employees	1	2	3	4	5
6	Please circle nb. 4, this is a checkpoint	1	2	3	4	5
7	The superior's personal likings do not affect disturbingly to his/her decisions	1	2	3	4	5
8	Our superior treats his/her employees friendly and considerately	1	2	3	4	5
9	Our superior can be trusted	1	2	3	4	5

46. The following questions are about your input on your work and how rewarding your work is. Circle the alternative which describes your current situation the best.

		**very little**	**little**	**not little nor much**	**much**	**very much**
1	How much do you feel that you make an input of your talent and strength on your work?	1	2	3	4	5
2	How much value for your work do you get as income, advantages e.g.?	1	2	3	4	5
3	How much value for your work do you get as acknowledgement and appreciation?	1	2	3	4	5
4	How much value for your work do you get as personal satisfaction?	1	2	3	4	5

47. Do you get/ have you got enough introduction for your work from your superior or co-workers?

1 not enough
2 quite enough
3 enough

48. Does your current work correspond to your education?

1 not at all
2 somewhat
3 yes, fully

49. Does your current job promote your professional career planning?

1 not at all
2 quite little
3 quite much
4 very much

50. Next we ask you about some factors connected to your work environment. Circle the right alternative.

		**daily**	**weekly**	**occasionally**	**never**
1	Do you have to work at uncomfortable working positions?	1	2	3	4
2	Does your work include repetitious, monotonous movements?	1	2	3	4
3	Does your work include lifting, carrying or holding by hands?	1	2	3	4
4	Do you have to work in a hot, cold or varying temperature?	1	2	3	4
5	Is there dust, smoky smell, gas, smoke or other factors weakening the air quality at your work?	1	2	3	4
6	Is there cigarette smoke due to other peoples’ smoking at your work?	1	2	3	4
7	Do you have to work in an inadequate lightning or dazzle?	1	2	3	4
8	At your work is there the smell of mold or underground cellar?	1	2	3	4
9	Do you have to use hazardous or dangerous chemicals at your work?	1	2	3	4
10	Is there a risk of accidents at your work?	1	2	3	4
11	Is there such a loud noise at your work that you cannot hear a normal speech within a meter’s reach?	1	2	3	4
12	At your work do you use shaking tools by hand or shaking machines?	1	2	3	4

13	Is there trembling directed to your whole body at your work?	1	2	3	4

51. Do the following factors bother your work? Circle the suitable alternative.

		**No or does not bother**	**Bothers somewhat**	**Bothers quite much**	**Bothers very much**
1	Noise	1	2	3	4
2	Dust	1	2	3	4
3	Trembling	1	2	3	4
4	Chemicals	1	2	3	4
5	Gas	1	2	3	4
6	Cigarette smoke	1	2	3	4
7	Cold	1	2	3	4
8	Heat	1	2	3	4
9	Inadequate lighting	1	2	3	4
10	Molds	1	2	3	4
11	Uncomfortable working positions	1	2	3	4
12	Monotonous work movements	1	2	3	4
13	Lifting, carrying, holding	1	2	3	4

52. This is where the actual questions end. We kindly ask you to check that you have answered each question according to the given instructions.

Was the answering?

1 easy
2 somewhat easy
3 somewhat difficult
4 difficult

Thank you for completing the questionnaire!

While completing this questionnaire if some topics have come to your mind of which you want to tell more or, if you want to comment this questionnaire, please write your question/comments below.
